# Serotype diversity and risk factors for pneumococcal carriage among healthy children in Klang Valley, Malaysia: A pre-vaccination cross-sectional study

**DOI:** 10.1016/j.ijregi.2025.100814

**Published:** 2025-11-25

**Authors:** Revathy Arushothy, Cheng Ngee Tan, Nur Asyura Nor Amdan, Mohammad Ridhuan Mohd Ali, Ratna Mohd Tap, Prem Ananth Paliappan, Yii Ling Liow, Saraswathiy Maniam, Salina Mohamed Sukur, Rohaidah Hashim

**Affiliations:** 1 Institute for Medical Research, National Institutes of Health Malaysia, Ministry of Health, Setia Alam, Selangor, Malaysia; 2 Institute of Biological Sciences, University Malaya, Kuala Lumpur, Malaysia; 3 Unit of Microbiology, Department of Pathology, Hospital Sultanah Aminah Johor Bahru, Johor Bahru, Johor, Malaysia

**Keywords:** *Streptococcus pneumoniae*, Pneumococcal carriage, Serotype distribution, Risk factors, Children, Malaysia

## Abstract

**Objectives:**

Carriage of *Streptococcus pneumoniae* among children aged <5 years facilitates transmission and invasive pneumococcal disease (IPD) progression, contributing to global childhood morbidity and mortality. Pneumococcal conjugate vaccines (PCVs) can reduce disease and carriage prevalence, though their effectiveness varies by regional serotype distribution. Malaysia introduced PCVs into the National Immunization Program in late 2020, but data on pneumococcal carriage and risk factors in children before vaccine implementation remain limited. This study aims to identify pneumococcal carriage, diversity, and risk factors among the unvaccinated children in Klang Valley, Malaysia.

**Methods:**

A cross-sectional study conducted from August 2018 to May 2019 involved 101 healthy children aged 2-5 years from 30 childcare centers in Klang Valley, Malaysia. Oropharyngeal swabs, which are more acceptable for young children although less sensitive than nasopharyngeal swabs for pneumococcal detection, were collected and enriched in Todd Hewitt Broth before DNA extraction. Carriage was identified using real-time polymerase chain reaction (PCR) targeting *lytA* gene, with positive samples serotyped using conventional multiplex PCR. Demographic and environmental data from structured questionnaires were analyzed for associations with pneumococcal carriage and multiple serotype colonization using logistic regression, chi-square, and Fisher exact test.

**Results:**

Pneumococcal carriage was detected in 39.6% (n = 40) of children, with 33 distinct serotypes identified. Both vaccine serotypes (VTs) and non-VTs were detected, with 67.5% of carriers colonized by multiple serotypes. Household size >5 members was significantly associated with carriage (adjusted odds ratio 4.62, 95% confidence interval 1.07-23.10, *P* = 0.047), while no demographic, behavioral, or environmental factors were significantly associated with multiple serotype colonization. VTs detected (19F, 6A/6B, 3, 1, and 5) were previously associated with IPD in Malaysia.

**Conclusions:**

This study provides crucial pre-PCV baseline data on pneumococcal carriage, serotype diversity, and risk factors in children of Klang Valley, Malaysia. The high prevalence of NVTs and frequent co-colonization highlight the need for ongoing surveillance to detect serotype replacement and guide evaluation of higher-valency PCVs. These findings can inform national immunization policy and strengthen strategies for monitoring vaccine impact in Malaysia.

## Highlights

• Overall, 39.6% of children had pneumococcal carriage pre-pneumococcal conjugate vaccine (PCV) vaccination.
• Household size of more than five significantly influenced pneumococcal carriage.
• A total of 67.5% of children carry multiple serotype pre-PCV vaccination.
• Non-vaccine serotypes predominantly colonize unvaccinated children in Klang Valley.

## Introduction

*Streptococcus pneumoniae* is a major cause of morbidity and mortality in young children worldwide, contributing to a wide spectrum of diseases ranging from non-invasive conditions such as otitis media and sinusitis to life-threatening invasive pneumococcal disease (IPD), including pneumonia, septicemia, and meningitis [1]. According to the World Health Organization (WHO), pneumococcal disease causes more than 300,000 deaths annually in children aged <5 years, with the highest burden occurring in low- and middle-income countries [2]. Carriage of *Streptococcus pneumoniae* in the upper respiratory tract, particularly the nasopharynx and oropharynx, serves as a necessary precursor to disease and plays a critical role in transmission dynamics within communities. Carriage is particularly common in children aged <5 years, who serve as the primary reservoirs due to their close contact in group settings, underdeveloped immune systems, and frequent exposure to respiratory infections [3,4]. A recent systematic review from Southeast Asia reported a pooled pneumococcal carriage prevalence of 36% among children aged <5 years with an estimated 7.6% prevalence in Malaysia [3]*.*

PCVs have been shown to reduce both IPD and carriage of vaccine-type serotypes in multiple countries [5]. In Malaysia, pneumonia accounted for 3.8% of deaths among children aged <5 years in 2016 and was the third leading cause of infant mortality in 2019 [6,7]. To mitigate the burden of pneumococcal infections toward children, PCVs were introduced into the National Immunization Program (NIP) in December 2020 starting with PCV10 [8]. Although national IPD surveillance data are limited, local studies have shown that *Streptococcus pneumoniae* is a major contributor to severe childhood pneumonia and invasive disease [9,10]. Before national introduction, PCV use was largely restricted to the private medical sectors, and small-scale pneumococcal carriage studies together with limited IPD serotype surveillance data were used as proxies to understand circulating serotypes and anticipate vaccine impact [8]. Establishing robust pre-vaccination baseline data is therefore critical for monitoring serotype replacement and guiding evidence-based vaccine policy in Malaysia.

Several risk factors have been associated with pneumococcal carriage globally, including young age, household crowding, and exposure to respiratory infections [4]. Despite the significance of pneumococcal carriage in disease transmission and vaccine planning, Malaysia has limited published data on colonization among healthy children, particularly in community settings. Invasive disease surveillance is essential for assessing clinical impact, whereas carriage studies provide further insights into prevalent serotypes within the community, enabling early detection of probable serotype shifts following vaccine introduction. In addition, local data on potential risk factors such as household size, environmental exposures, and socioeconomic conditions are limited. The extent of vaccine effectiveness also varies depending on regional serotype distribution, which is influenced by demographic, environmental, and socioeconomic factors.

This study aims to determine the prevalence, risk factors, and serotype diversity associated with pneumococcal carriage among children attending childcare centers in Klang Valley, before the implementation of pneumococcal vaccination in the NIP. These baseline data are intended to strengthen understanding of pneumococcal epidemiology in Malaysia and provide a foundation for future surveillance and vaccine impact assessment at the national level.

## Methods

### Study site and population

This was a cross-sectional study conducted among healthy children attending childcare centers in Klang Valley, Malaysia. Participants were prospectively enrolled and sampled between from August 2018 to May 2019. However, laboratory analyses of the collected samples were only conducted in 2024.

This research concentrated on Klang Valley, an urban area in Malaysia, reflecting the demographic specificity of this location rather than the whole country. A total of 30 registered childcare centers were selected using convenience sampling. Centers were identified through local childcare networks (registered under the Ministry of Education, Malaysia) and approached for participation. Recruitment was carried out through a formal invitation letter issued by the Director of the Institute for Medical Research (IMR). Healthy children aged between 2 and 5 years who were enrolled in these centers were invited to participate upon approval. Parents were invited to enroll their children if their children met the inclusion criteria. All 30 daycare centers that took part in the study submitted in samples. However, the number of children per center varied based on parental consent and the center’s participation rates. Records of centers that declined participation were not systematically captured, and therefore, selection bias cannot be excluded, which may limit the generalizability of the findings.

Inclusion criteria were children aged 2 to 5 years, with written informed consent obtained from parents or legal guardians. Exclusion criteria included the presence of acute respiratory symptoms within 72 hours before enrollment, antibiotic consumption within the past 7 days, known immunodeficiency, any physical condition deemed unsuitable for oropharyngeal swab collection, or lack of parental consent. Ethical approval was obtained from the Malaysian Medical Research and Ethics Committee (MREC), Ministry of Health (reference: KKM/NIHSEC/P18-876(11)). Before sampling, parents or guardians provided written informed consent and completed a structured questionnaire. All specimens were obtained before the initiation of the Malaysian National Pneumococcal Vaccination Program, and none of the enrolled children had received pneumococcal conjugate vaccination at the time of sampling.

### Sampling and data collection

A total of 30 childcare centers consented to participate in the study. Information sheets detailing the study objectives and procedures, along with structured questionnaires, were distributed to parents or legal guardians. The questionnaire was available in both English and Malay and included independent variables such as vaccination status, health history, socioeconomic background, demographic details, and environmental exposures. Anthropometric measurements, including weight and height, were also recorded.

Following the return of completed questionnaires and written informed consent, oropharyngeal swabs were collected from participating children by trained healthcare professionals. The use of oropharyngeal swabs was in line with WHO consensus for pneumococcal carriage studies in young children, as this method is less invasive and more acceptable while still providing reliable detection. The swabs were placed in Amies transport medium containing charcoal (Vacutest Kima, Italy) and sent to the Bacteriology Unit, IMR, Kuala Lumpur, under cold-chain conditions (2-8°C) within 24 hours of collection for further processing. Upon arrival at the laboratory, the swabs were transferred into Todd Hewitt Broth (THB) supplemented with 15% (v/v) glycerol and stored at –80°C for long-term preservation until subsequent microbiological analysis.

### Pneumococcal enrichment and DNA extraction

A 200 µl aliquot of the thawed oropharyngeal swab suspension in THB with 15% (v/v) glycerol was enriched by transferring it into 1.5 ml of fresh THB supplemented with 100 µL of rabbit serum. The mixture was incubated overnight at 37°C in a CO₂-enriched atmosphere to promote pneumococcal growth. The enriched culture was then centrifuged at 10,000 × g for 10 minutes. The supernatant was discarded, and the resulting pellet was used for genomic DNA extraction using the DNeasy UltraClean Microbial Kit (Qiagen, Germany), following the manufacturer’s protocol.

### Pneumococcal carriage screening

Real-time polymerase chain reaction (RT-PCR) targeting the *lytA* gene was used to screen for pneumococcal carriage, as *lytA* is a well-established molecular marker for *Streptococcus pneumoniae* identification [11]. Each 20 µL reaction mixture contained 2× Promega GoTaq™ Probe qPCR Master Mix (Promega Corporation, USA), 200 nM of each primer (*lytA*-F and *lytA*-R), 200 nM probe, and molecular-grade water. Amplification was performed using the CFX96 Touch RT-PCR Detection System (Bio-Rad Laboratories, USA).

Thermal cycling conditions consist of initial denaturation at 95°C for 10 minutes, followed by 40 cycles of denaturation at 95°C for 15 seconds and annealing/extension at 60°C for 15 seconds [11]. A standard curve was generated using a five-point serial dilution of known *S. pneumoniae* DNA concentrations, plotting cycle threshold (Ct) values against the logarithmic DNA concentration. Fluorescence drift correction was applied, and the relative fluorescence unit (RFU) threshold was set at 100. A Ct value of <40 was considered a positive result for *lytA* detection, indicating pneumococcal carriage. Appropriate positive control ATCC 49619 and negative control ATCC 25922 were included in each PCR run to ensure assay specificity, and samples with equivocal results were re-tested to confirm carriage status.

### Pneumococcal serotyping by sequential multiplex PCR

DNA extracted from oropharyngeal swabs that tested positive for the *lytA* gene was subjected to conventional sequential multiplex PCR for pneumococcal serotyping [12]. The sequential multiplex PCR assays followed the Centers for Disease Control and Prevention (CDC)-recommended protocol, which together cover most globally and regionally relevant serotypes, including those reported in Malaysia. All primer sets, amplification conditions, and band-size references for serotype identification followed the CDC sequential multiplex PCR protocol, and no new or modified band patterns were developed in this study. Each PCR reaction was optimized and contained 2 µl of template DNA, 12.5 µl of 2× MyTaq Red Mix (Meridian Bioscience, USA), varying volumes of multiplex primer sets as described in the protocol, and molecular-grade water to a final volume of 25 µl. Amplification was performed using the Mastercycler® nexus X2 (Eppendorf, Germany) under the following cycling conditions: initial denaturation at 95°C for 4 minutes; followed by 35 cycles of denaturation at 95°C for 45 seconds, annealing at 54°C for 45 seconds, and extension at 65°C for 2 minutes and 30 seconds. All runs included positive control ATCC 49619 and negative control ATCC 25922 for quality assurance.

Following amplification, 4 µl of each PCR product was loaded onto a 2.0% agarose gel alongside a 100 bp DNA ladder (GeneDireX, USA). Electrophoresis was carried out at 100 V and 3 A for 40 minutes. Gels were visualized under UV light using the GelDoc Go Gel Imaging System (Bio-Rad, USA), and individual serotypes or serogroups were identified based on DNA band sizes, interpreted according to the CDC protocol.

### Data analysis

Data from questionnaires and laboratory results were compiled and exported to Microsoft Excel, then analyzed using GraphPad Prism version 10.3.1 for Windows (GraphPad Software, Boston, Massachusetts USA, www.graphpad.com). With 101 participants from Klang Valley, the prevalence estimate of *Streptococcus pneumoniae* carriage (39.6%) had a 95% CI width of approximately ± 9.5%, sufficient for a local baseline estimate. Associations between pneumococcal carriage and potential risk factors were analyzed using univariate analysis, including simple logistic regression, chi-square tests, and Fisher’s exact tests, as appropriate. Results were presented as odds ratio (OR) with 95% CI. All variables with *P*-value <0.25 were included in multivariate logistic regression model to identify factors independently associated with carriage. A *P*-value <0.05 in multivariate analysis was considered statistically significant. Additionally, similar analytical approaches were used to examine risk factors that potentially contributes to concurrent colonization with multiple pneumococcal serotypes among carriers in the study. Model fit and collinearity were checked and found acceptable. Records with missing data were excluded from multivariate analyses but included in descriptive analyses.

## Results

### Risk factors associated with Streptococcus pneumoniae carriage

Among the 101 children included in the study, 40 (39.6%) exhibited pneumococcal carriage as determined by *lytA* gene detection (Table 1). Univariate analysis was conducted to assess associations between pneumococcal carriage and various demographic, socioeconomic, and environmental factors. Results depicted that gender, race, living density, basic sanitation practices, average household income, history of traveling abroad, body mass index, habit of sharing cups, bath frequency, and the number of people sharing room were found insignificant to affect pneumococcal carriage among healthy children in Klang Valley. Based on univariate analysis, household size >5 members (*P* = 0.05), age of 2 years (*P* = 0.13), the habit of sharing utensils (*P* = 0.18), and sharing a room with ≥4 people were identified as variables meeting the inclusion threshold (*P* <0.25) for multivariate analysis (Table 2). Only household size remained statistically significant (*P* <0.05) in the final model (adjusted OR 4.62; 95% CI, 1.07-23.10, *P* = 0.047). There was no statistically significant association with carriage observed for respondents aged 2 years, sharing spoon, fork and knives, or sharing a room with >4 people.

**Table 1:** Univariate analysis of risk factors that influence the carriage of *Streptococcus pneumoniae* among children attending childcare centers in Klang Valley, Malaysia.

	Total Children, n (%)	*S. Pneumoniae* Positive carrier, n (%)	*S. Pneumoniae* Negative carrier, n (%)	Odds ratio (95% Cl)	*P*
Age					
2	28 (27.7)	8 (28.6)	20 (71.4)	0.46 (0.16, 1.22)	0.13^a^
3	28 (27.7)	11 (39.3)	17 (60.7)	0.74 (0.28-1.92)	0.54^a^
4	45 (44.6)	21 (46.7)	24 (53.3)	Ref	
Gender					
Male	53 (52.5)	23 (43.4)	30 (56.6)	0.72 (0.33-1.64)	0.41^c^
Female	48 (47.5)	17 (35.4)	31 (64.6)	Ref	
Nationality					
Malaysian	98 (97.0)	40 (40.8)	58 (59.2)	Na	Na
Non-Malaysian	3 (3.0)	0 (0.0)	3 (100.0)		
Race					
Malay	80 (79.2)	29 (36.3)	51 (63.7)	1.45 (0.64-3.21)	0.39^c^
Non-Malay	21 (20.8)	11 (52.4)	10 (47.6)	Ref	
					
Living area					
High-density	67 (66.3)	25 (37.3)	42 (62.7)	1.57 (0.65-3.67)	0.32^c^
Low-density	29 (28.7)	14 (48.3)	15 (51.7)	Ref	
No data	5 (5.0)	1 (20.0)	4 (80.0)		
Household Size					
<5	41 (40.6)	11 (26.8)	30 (73.2)	Ref	
>5	54 (53.5)	25 (46.3)	29 (53.7)	0.43 (0.18-0.99)	0.05^c^
No data	6 (5.9)	4 (66.7)	2 (33.3)		
Source of water supply					
Treated water	93 (92.1)	35 (37.6)	58 (62.4)	NA	
Well	2 (2.0)	2 (100.0)	0 (0.0)		
No data	6 (5.9)	3 (50.0)	3 (50.0)		
Basic sanitation					
Flush or dry toilet	94 (93.1)	37 (39.4)	57 (60.6)	NA	
Proper sewage	82 (81.2)	33 (40.2)	49 (59.8)	2.23 (0.43-12.96)	0.65^b^
Solid waste disposal	79 (78.2)	30 (38.0)	49 (62.0)	0.20 (0.02-1.45)	0.30^b^
Average household income					
Low income (<Rm3000)	62 (61.4)	25 (40.3)	37 (59.7)	0.93 (0.37-2.26)	0.87^c^
Middle/high income (>Rm3000)	26 (25.7)	10 (38.5)	16 (61.5)	Ref	
No data	13 (12.9)	5 (38.5)	8 (61.5)		
History of travelling abroad	16 (15.8)	6 (37.5)	10 (62.5)	1.12 (0.39-3.50)	0.84^c^
BMI for age					
Normal	33 (32.7)	16 (48.5)	17 (51.5)	Ref	
Underweight	9 (8.9)	4 (44.4)	5 (55.6)	0.85 (0.18-3.77)	0.83^a^
Overweight	8 (7.9)	2 (25.0)	6 (75.0)	0.35 (0.05-1.80)	0.83^a^
No data	51 (50.5)	18 (35.3)	33 (64.7)		
Sharing utensils and cups					
Sharing spoon, fork, and knife	70 (69.3)	26 (37.1)	44 (62.9)	1.81 (0.73-4.13)	0.18 ^C^
Sharing cups and glass	70 (69.3)	27 (38.6)	43 (61.4)	1.22 (0.53-2.93)	0.66 ^C^
Sharing towels	23 (22.8)	9 (39.1)	14 (60.9)	0.93 (0.38-2.34)	0.89^c^
Frequency of bathing in a day					
<2	5 (5.0)	2 (40.0)	3 (60.0)	1.00 (0.20-5.84)	>0.99^c^
≥2	95 (94.1)	38 (40.0)	57 (60.0)	Ref	
No data	1 (0.9)	0 (0.0)	1 (100.0)		
No. Of people sharing room					
<2	10 (9.9)	6 (60.0)	4 (40.0)	Ref	
2-3	50 (49.5)	22 (44.0)	28 (56.0)	0.52 (0.12-2.06)	0.36^a^
>4	26 (25.7)	9 (34.6)	17 (65.4)	0.35 (0.07-1.55)	0.18^a^
No data	15 (14.9)	3 (20.0)	12 (80.0)		

a Simple logistic regression.

b Chi-square test.

c Fisher exact test.

**Table 2:** Multivariate analysis of risk factors that influence the carriage of *Streptococcus pneumoniae* among children attending childcare centers in Klang Valley, Malaysia.

	Total children, N (%)	Odds ratio (95% CI)	*P*	Adjusted odds ratio (95% CI)	*P*
**Age**					
**2**	28	1.48 (0.90, 2.50)	0.13^a^	0.60 (0.10-3.18)	0.56
**Household Size**					
**>5**	54	0.43 (0.18-0.99)	0.05^a^	4.62 (1.07-23.10)	0.047^d^
**Sharing utensils and cups**					
**Sharing spoon, fork, and knife**	70	1.81 (0.73-4.13)	0.18 c	0.57 (0.11-2.69)	0.39
**No. of people sharing room**					
**>4**	26	0.35 (0.07-1.55)	0.18^a^	1.52 (0.28-9.72)	0.64

a Simple logistic regression. ^b^Chi-square test.

c Fisher exact test.

d Reached statistical significance.

### Factors associated with multiple Pneumococcal serotype carriage

Among the 40 children identified with *Streptococcus pneumoniae* carriage, 23 distinct serotypes were detected. Of these, 13 children (32.5%) carried a single serotype, while 27 (67.5%) harbored multiple serotypes, defined as samples yielding more than one distinct serotype- or serogroup-specific band pattern. Univariate analysis was conducted to examine associations between multiple serotype carriage and various demographic, socioeconomic, and environmental factors (Table 3).

**Table 3:** Univariate analysis of risk factors associated with multiple serotype carriage of *Streptococcus pneumoniae* among children attending childcare centers in Klang Valley, Malaysia.

	Total Children, N	Total Samples With 1 Serotype Detected	Total Samples Detected More Than 1 Serotype	Univariable Analysis Or (95% Cl)	*P*
**Age**					
2	8	1	7	3.23 (0.42-67.49)	0.32^a^
3	11	5	6	0.69 (0.14-3.56)	0.65^a^
4	21	7	14	Ref	
**Gender**					
Male	23	5	18	0.25 (0.06-0.98)	0.05^c^
Female	17	9	8	Ref	
**Nationality**					
Malaysian	40	13	27	Na	
Non-Malaysian	0	0	0		
**Race**					
Malay	29	8	21	2.19 (0.51-8.68)	0.28^c^
Non-Malay	11	5	6	Ref	
**Living area**					
High-density	25	9	16	0.99 (0.26-4.12)	0.99^c^
Low-density	14	5	9	Ref	
No data	1	1	0		
**Household size**					
<5	11	5	6	Ref	
>5	25	6	19	2.64 (0.56-12.44)	0.20^b^
No data	4	2	2		
**Source of water supply**					
Treated water	35	14	21	Na	
Well	2	2	0		
No data	3	3	0		
B**asic sanitation**					
Flush or dry toilet	37	13	24	Na	
Proper sewage	31	9	22	2.23 (0.43-12.96)	0.65^c^
Solid waste disposal	30	10	20	0.20 (0.02-1.45)	0.30^c^
**Average household income**					
Low income (<Rm3000)	25	7	18	1.71 (0.43-8.04)	0.69^c^
Middle or high income (> Rm3000)	10	4	6	Ref	
No data	5	3	2		
**History of travelling abroad**	6	5	1	0.058 (0.005-0.46)	0.0089^b^
**BMI for age**					
Normal	14	5	9	Ref	
Underweight	4	2	2	0.63 (0.06-6.63)	0.68^b^
Overweight	2	1	1	0.5 (0.02-14.92)	0.65^b^
No data	17	3	14		
**Sharing utensils and cups**					
Sharing spoon, fork, and knife	26	7	19	2.33 (0.65-8.51)	0.23^c^
Sharing cups and glass	27	8	19	1.70 (0.41-6.19)	0.46^c^
Sharing towels	9	5	4	0.36 (0.10-1.75)	0.24^b^
**Frequency of bathing in a day**					
<2	2	1	1	Ref	
≥2	38	13	25	2.6 (0.13-50.98)	0.50^b^
No data	0	0	0		
**No. Of people sharing room**					
<2	6	1	5	Ref	
2-3	22	10	12	0.34 (0.02-2.74)	0.37^a^
>4	9	7	2	0.70 (0.03-9.48)	0.79^a^
No data	3	1	2		

a Simple logistic regression.

b Chi-square test.

c Fisher exact test.

Based on the univariate analysis, the risk factors such as age, race, living density, basic sanitation practices, average household income, body mass index, sharing cups and glasses, frequency of bath, and sharing room with two to three people were found not significant to influence the carriage of >1 serotype among children. The independent variables found significant (*P ≤* 0.25) where respondent were male gender, household size >5 members, history of traveling abroad, habits of sharing utensils, and sharing towels. Subsequent standard multiple logistic regression analysis (Table 4) found these variables were not statistically significant for influencing multiple serotype carriage. Given the small number of events, these estimates are exploratory and interpreted in the context of the Malaysian household environment.

**Table 4:** Multivariate analysis of risk factors associated with multiple serotype carriage of *Streptococcus pneumoniae* among children attending childcare centers in Klang Valley, Malaysia.

	Total children, N (%)	Odds ratio (95% CI)	*P*	Adjusted odds ratio (95% CI)	*P*
**Gender**					
**Male**	23	0.25 (0.06-0.98)	0.053^c^	6.75 (0.83-87.10)	0.10
**Household size**					
**>5**	25	2.64 (0.56-12.44)	0.20^b^	2.71 (0.23-30.26)	0.41
**History of travelling abroad**	6	0.058 (0.005-0.46)	0.0089^b^	0.01 (0.002-1.8)	0.15
**Sharing utensils and cups**					
**Sharing spoon, fork, and knife**	26	2.33 (0.65-8.51)	0.23^c^	3.83 (0.44-41.00)	0.22
**Sharing towels**	9	0.36 (0.10-1.75)	0.24^b^	1.11 (0.07-30.73)	0.94

^a^Simple logistic regression

b Chi-square test.

c Fisher exact test.

### Serotype distribution and estimated vaccine coverage

**Figure 1:**
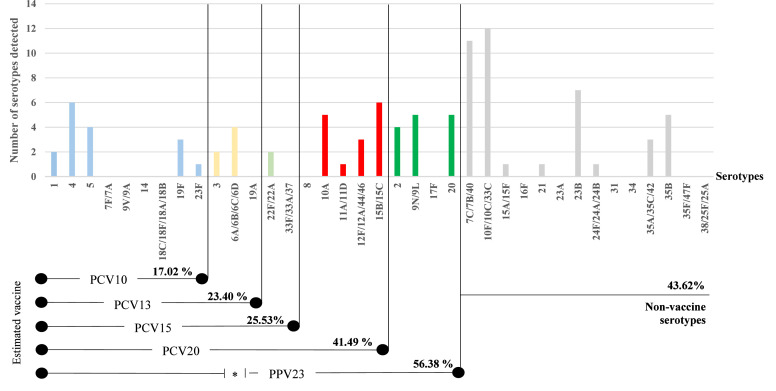
Distribution of serotypes in carriage of Streptococcus pneumoniae among children attending childcare centers in Klang Valley, Malaysia Serotypes are color-coded by vaccine inclusion: light blue (PCV10), yellow (PCV13), light green (PCV15), red (PCV20), green (PPV23), and grey (non-vaccine types). PPSV23 range is marked with an asterisk (*) because it does not cover serogroup/serotypes 6. PCV, pneumococcal conjugate vaccine; PPV, pneumococcal polysaccharide vaccine.

A total of 23 distinct serotypes or serogroups were detected among children with pneumococcal carriage in the study cohort, indicating substantial serotype diversity (Figure 1). Several vaccine-type (VT) serotypes targeted by pneumococcal vaccines currently used in Malaysia (PCV10 and PCV13) were identified. The most frequently detected serotype was serotype 4 (15.0%), followed by serotypes 5 (10.0%), 6A/6B/6C/6D (10.0%), 19F (7.5%), 3 (5.0%), 1 (5.0%), and 23F (2.5%). In addition, several prevalent serotypes with extended vaccine coverage were observed. These included 22F/22A (5.0%), covered by PCV15, and serotypes 15B/15C (15.0%), 10A (12.5%), 12F/12A/44/46 (7.5%), and 11A/11D (2.5%), which are included in PCV20. Serotypes targeted by the 23-valent pneumococcal polysaccharide vaccine (PPV23) were also common, particularly serotypes 9N/9L (12.5%), 20 (12.5%), and 2 (10.0%).

Non-VTs (NVT) accounted for 43.6% of the pneumococcal isolates. The most frequently detected NVTs were serogroups 10F/10C/33C (30.0%) and 7C/7B/40 (27.5%). Other identified NVTs included 23B (17.5%), 35B (12.5%), 35A/35C/42 (7.5%), 15A/15F (2.5%), 21 (2.5%), and 24F/24A/24B (2.5%).

Estimated vaccine coverage varied by formulation (Figure 1). The 10-valent PCV (PCV10) covered 17.0% of isolates, including serotypes 1, 4, 5, 19F, and 23F. Coverage increased to 25.5% with PCV13 through the inclusion of serotypes 6A/6B and 3. PCV15, which includes serotype 22F, increased estimated coverage to 28.3%; however, serotype 33F was not detected in this cohort. PCV20 further expanded coverage to 41.5% by including additional serotypes such as 10A, 11A, 12F, and 15B. The 23-valent pneumococcal polysaccharide vaccine (PPV23), typically administered to adults, provided the highest estimated coverage at 56.4%, due to the inclusion of additional serotypes such as 2, 9N, 20, and 35B.

## Discussion

This study provides a comprehensive overview of pneumococcal carriage, serotype diversity, and associated risk factors among healthy children attending childcare centers in Klang Valley, Malaysia, before the implementation of PCVs in the NIP. The findings offer important baseline data for assessing the future impact of vaccination efforts in the country.

The overall pneumococcal carriage rate observed in this study was 39.6%, which is comparable to regional data from Southeast Asia, reported at 36.0% [3]. In Malaysia, earlier studies documented carriage rates of 35.4% in 2013 [13] and 22.6% in 2025 [14]. Although this study was limited to children from only 30 childcare centers in Klang Valley, the high carriage rate suggests substantial pneumococcal circulation in urban preschool populations. Currently, Malaysia has limited pneumococcal carriage data, especially from healthy children. Most available information comes from invasive disease surveillance, which does not reflect asymptomatic carriage or the full serotype diversity circulating in the community. Carriage studies offer critical insights into the circulating strains both pre- and post-vaccination, which helps identify early trends in emerging NVTs, evaluate vaccine impact, and guide future immunization strategies.

Multivariate analysis found that children from households with more than five members were more likely to carry pneumococci, although there is considerable uncertainty in this estimate, likely due to the limited number of children in larger households. This observation aligns with global evidence linking crowded living conditions to increased transmission, particularly among children aged <5 years [4,15]. To date, published data from Malaysia on pneumococcal carriage in healthy children remain limited, with most studies focusing on invasive disease or antimicrobial resistance. Our findings underscore the role of the household environment in sustaining pneumococcal circulation, particularly in culturally common multigenerational homes. In such settings, close contact between young children and elderly family members may facilitate bidirectional transmission. These observations support consideration of broader vaccination strategies that include adults and older individuals at risk. Enhancing household-level immunity through vaccination could help reduce transmission and support overall herd protection [16].

Furthermore, this study highlights the high serotype diversity of *Streptococcus pneumoniae* isolates from healthy children in the pre-vaccine era. A total of 23 distinct serotypes or serogroups were identified, reflecting substantial pneumococcal heterogeneity and suggesting ongoing transmission of a wide range of serotypes within the community. Similar observations have been reported in a pneumococcal carriage study from Tunisia and a meta-analysis from Latin America, both of which identified many serotypes among healthy children, including an increasing prevalence of NVTs [17,18]. Several VTs were detected, with serotype 4 being the most common (15.0%), followed by serotype 5 (10.0%), serogroup 6A, 6B, 6C, and 6D (10.0%), and serotype 19F (7.5%). Despite being included in current pneumococcal vaccines, these VTs were not predominant among children with carriage. In contrast, NVTs were more prevalent, particularly serogroups 10F, 10C, and 33C (30.0%) and 7C, 7B, and 40 (27.5%), which are not targeted by existing PCV formulations. A recent study by Mohamad et al. in 2025 also found NVTs to be the most frequently detected serotypes, specifically 15C and 23A, among Malaysian children from 2023 to 2024 [11]. Although dominant NVTs varied across studies, their consistent presence highlights their potential role in serotype replacement, where NVTs occupy the ecological space left by vaccine-targeted serotypes following immunization. This pattern has also been observed in several high-income countries, where different NVT profiles emerged after the introduction of PCVs [19].

The detection of pneumococcal serotypes 3, 19F, and the 6A/6B/6C/6D serogroup among healthy children in this study indicates a risk of progression to invasive disease. Previous studies in Malaysia and across Southeast Asia have reported 6B and 19F as among the most common serotypes causing pediatric IPD, and other serotypes such as 1, 14, and 19A were also commonly associated with invasive disease in Malaysia, with 6B and 14 particularly prevalent among healthy children [3,9,20,21]. Serotype 14 is considered a poor colonizer but exhibits high virulence and a strong propensity to cause illness such as IPD, particularly in children, and this characteristic is reflected in our cohort, where no serotype 14 isolates were detected among healthy carriers. Apart from this, the absence of serotype 14 in this cohort suggests that the regional carriage distribution observed here may not fully represent the serotype patterns or the IPD burden across all Malaysian regions. Local studies also reported a higher proportion of VT pneumococcal carriage in the Peninsula (69.5% by PCV13) as compared to East Malaysia (33.3% by PCV13). However, an estimated high coverage of VTs up to 88.2% by PCV13 suggested a reduction in pneumococcal disease burden following vaccine rollout [9,21]. This is further supported by a post-vaccination assessment in Singapore, reporting a decrease in IPD incidence and PCV-covered serotypes [22]. Despite this broad coverage by pneumococcal vaccines available in Malaysia (PCV10 and PCV13), the circulation of VTs in carriage population highlights the need for ongoing surveillance and revision of preventive strategies.

A notable finding in this study is the prevalence of serogroup 15B/15C (15.0%) and serotype 10A (12.5%), which are only incorporated in high-valent PCV20. Although these NVTs were common in carriage, their predicted invasiveness is considered low to moderate, suggesting a limited contribution to severe disease. In contrast, the detection of serogroup 9N/L (12.5%), serotype 20 (12.5%), and 2 (10.0%), all of which are covered by the 23-pneumococcal polysaccharide vaccine (PPV23), raise concerns for pediatric populations. While PPV23 offers the widest serotype-specific coverage, it is not recommended for children due to limited T-cell-independent immune response, no generation of immune memory, and the potential of immune hypo-responsiveness in subsequent immunization. However, PPV23 use in older adults can reduce disease burden and may indirectly confer herd protection in children, particularly in large households where intergenerational transmission is common. This observation reinforces the requirement of higher-valency conjugate vaccines to include PPV23-covered serotypes [23].

PCV10 vaccine coverage was 17.0% among healthy children carrying *Streptococcus pneumoniae*. The inclusion of additional serotypes in PCV13 and PCV15 increased the potential coverage to 25.5% and 28.3%, respectively. PCV20 demonstrated the highest coverage among the conjugate vaccines, targeting 41.5% of the serotypes detected in this study. The broadest overall serotype coverage was observed with the 23-valent pneumococcal polysaccharide vaccine (PPV23), which covered up to 56.4% of the detected serotypes. However, PPV23 is not recommended for children despite its broad serotype coverage, due to its poor immune response generated against pneumococcal infections [8,23]. Although these estimates are based on carriage isolates, carriage is a key reservoir for transmission and progression to invasive disease, making these findings highly relevant for anticipating vaccine impact**.** In contrast, the inclusion of higher-valency PCVs are endorsed in previous estimates due to a greater reduction of disease cases, deaths, and medical cost 5 years post-vaccination [24].

Analysis of risk factors influencing serotype prevalence revealed that 67.5% of pneumococcal carriage cases involved multiple serotypes, likely reflecting high exposure and complex colonization dynamics of *Streptococcus pneumoniae*. While most tested variables were not statistically significant, certain trends were observed. Children from larger households, males, and those who shared utensils appeared more likely to carry multiple serotypes, consistent with the role of crowding and close contact in facilitating transmission. Although limited by the small sample size and wide CIs, these patterns are biologically plausible and align with findings from other settings where household structure and child-to-child contact increased carriage risk. These patterns are biologically plausible and align with evidence from other settings where household structure and child-to-child contact increased carriage risk. The study’s geographical scope is restricted to Klang Valley, which may influence the findings. Besides, the study population of 101 respondents are not enough to represent the national population, which possibly leads to a biased statistical outcome. Nevertheless, these findings suggest that multiple serotype carriage may be influenced by both exposure dynamics within the environment and individual behavioral factors, especially in a multicultural close-limit family structure prevalent in Malaysian society.

This study has several limitations that should be considered in interpreting the findings. The sampling was limited to Klang Valley, an urban region, which may not accurately represent pneumococcal carriage patterns in rural or other parts of Malaysia. The use of convenience sampling in childcare centers also introduces the possibility of selection bias, as participating centers may not reflect the wider community. In addition, the use of oropharyngeal swabs, although a more acceptable sampling method for children, may lead to lower sensitivity compared to the WHO-recommended nasopharyngeal swabbing method, potentially leading to underestimation of true carriage prevalence [25]. However, although less sensitive than nasopharyngeal swabs, our observed carriage rate was consistent with other regional studies using nasopharyngeal samples, suggesting that detection in this study was not substantially affected by the sampling method. The cross-sectional and retrospective design also limit the ability to determine causal relationships and does not account for temporal factors such as seasonal variation. Additionally, the use of conventional multiplex PCR meant that multiple serotypes within a single serogroup could not be distinguished, potentially reducing serotype resolution. Lastly, vaccine coverage estimates were based on carriage data, providing insights into the potential impact of vaccines on circulating serotypes in the community. Assessment of protection against invasive disease would require complementary clinical surveillance data.

Future research should expand pneumococcal carriage surveillance to include both urban and rural populations across different regions of Malaysia. Longitudinal studies are needed to track serotype changes, vaccine impact, and antimicrobial resistance over time. Advanced molecular methods such as whole-genome sequencing may enhance detection of emerging clones. Integrating carriage data with invasive disease surveillance would provide a more comprehensive evaluation of vaccine effectiveness. Additionally, immunization strategies should consider extending protection to adults and the elderly, especially in multigenerational households, to strengthen community-level immunity. With PCV10 introduced nationally in the end of 2020 [8], ongoing surveillance is vital to assess shifts in serotype distribution, detect early signs of serotype replacement, and ensure optimal protection.

## Conclusion

This study provides important baseline data on pneumococcal carriage, serotype distribution, and associated risk factors among healthy children in Klang Valley, Malaysia, before the introduction of PCVs. These findings are valuable for public health authorities in guiding immunization strategies and tailoring vaccine implementation to local epidemiology. Understanding the prevalence of vaccine and NVTs, along with key risk factors, can support informed vaccine selection, help identify vulnerable groups, and address coverage gaps. As Malaysia transitions to newer and higher-valency PCVs, this baseline will serve as a reference point to evaluate vaccine impact, monitor changes in serotype dynamics, and assess the long-term effectiveness of the NIP in reducing pneumococcal disease burden.

## Declaration of competing interest

All inferences, opinions, and conclusion drawn in this manuscript are those of the authors and do not reflect the opinions or policies of the data steward(s). The authors declare that they have no known competing financial interests or personal relationships that could have appeared to influence the work reported in this paper.
